# Maladaptive perfectionism and experiential avoidance as transdiagnostic pathways between eating disorder and social anxiety symptoms in young adults

**DOI:** 10.1186/s40337-026-01614-w

**Published:** 2026-04-26

**Authors:** Rafael Triguero-Sánchez, María J. Triguero-López

**Affiliations:** 1 Pontificia Universidad Católica del Perú, CENTRUM Católica Graduate Business School, Lima, Peru; 2 Department of Personality, Assessment, and Psychological Treatment, Faculty of Psychology, University of Seville, Seville, Spain

**Keywords:** Eating disorder symptoms, Social anxiety, Maladaptive perfectionism, Experiential avoidance, Transdiagnostic processes, Young adults

## Abstract

**Background:**

Eating disorder (ED) and social anxiety (SA) symptoms frequently co-occur and are associated with greater clinical severity and poorer outcomes. From a transdiagnostic perspective, maladaptive perfectionism and experiential avoidance have been proposed as shared vulnerability processes, yet they have rarely been examined simultaneously within integrative structural frameworks.

**Methods:**

This study examined bidirectional associations between ED and SA symptom risk and evaluated the role of maladaptive perfectionism and experiential avoidance as transdiagnostic processes within a cross-sectional structural equation modelling framework. A non-clinical sample of 705 young adults aged 18–25 years was recruited from educational settings in Spain. Participants completed validated self-report measures assessing ED (Eating Attitudes Test-26, EAT-26; [28]) and SA symptom risk (Liebowitz Social Anxiety Scale, LSAS; [50] ), maladaptive perfectionism (concern over mistakes and personal standards) (Frost Multidimensional Perfectionism Scale, FMPS; [26]), and experiential avoidance (Personalized Psychological Flexibility Index, PPFI; [41]). Structural equation modelling with bootstrap estimation was used to estimate direct and indirect associations and to compare alternative models.

**Results:**

ED and SA symptom risk showed significant bidirectional associations. Maladaptive perfectionism was consistently associated with higher ED and SA symptom risk through direct and indirect associations. Experiential avoidance exhibited a more complex pattern, characterised by negative direct associations alongside positive indirect associations with both symptom domains. Models including both transdiagnostic processes demonstrated superior fit and parsimony compared with models excluding these processes or including a single process.

**Conclusions:**

These findings highlight maladaptive perfectionism and experiential avoidance as relevant transdiagnostic processes associated with the co-occurrence of ED and SA symptoms in young adults. The results support integrative, process-focused perspectives for understanding ED–SA comorbidity and may inform assessment and formulation within transdiagnostic frameworks.

## Abstract (plain language summary)

Eating disorder symptoms and social anxiety symptoms often occur together, especially during adolescence and young adulthood. When both types of symptoms are present, people often experience greater emotional distress and find everyday situations more challenging. However, the reasons why these difficulties are so closely linked are not yet fully understood.

This study explored whether two common psychological processes—maladaptive perfectionism (setting very high standards and being overly critical of mistakes) and experiential avoidance (trying to avoid unpleasant thoughts and emotions)—are associated with the co-occurrence of eating disorder and social anxiety symptoms. A large group of young adults completed questionnaires about eating-related concerns, social anxiety, perfectionism, and how they respond to difficult internal experiences.

The findings showed that eating disorder and social anxiety symptoms were strongly related to each other. Both maladaptive perfectionism and experiential avoidance were linked to higher levels of symptoms in both areas. Avoiding uncomfortable thoughts or feelings appeared to offer short-term relief but was also associated with greater difficulties through indirect pathways.

These results suggest that focusing on shared psychological processes, rather than on single diagnoses alone, may help improve understanding, assessment, and support for young people experiencing these difficulties.

## Background

Eating disorders (EDs) and social anxiety disorder (SAD) are highly comorbid conditions, particularly during adolescence and emerging adulthood, a developmental period characterised by heightened vulnerability to both disorders [61, 69]. Meta-analytic and epidemiological evidence indicates that social anxiety is one of the most prevalent anxiety conditions among individuals with eating disorders [30, 42], and that their co-occurrence is associated with greater symptom severity, poorer prognosis, higher chronicity, and poorer response to standard treatments [5, 71]. Longitudinal findings further suggest that social anxiety symptoms may prospectively increase the risk of developing eating disorder symptomatology, and vice versa [47]. Taken together, this evidence supports the view that ED and SAD symptoms may be embedded in overlapping systems of vulnerability rather than representing independent clinical entities, highlighting the need for transdiagnostic and dimensional approaches to understanding their comorbidity [49]. Although this body of work provides robust evidence of comorbidity, much of the literature has either focused on specific eating disorder diagnoses or has not consistently differentiated between restrictive and binge–purge presentations, despite evidence that partially distinct mechanisms may underlie these phenotypes [12, 58, 69]. Despite this robust evidence of bidirectional associations, the psychological processes sustaining ED–SAD comorbidity remain insufficiently specified [46].

In recent years, transdiagnostic approaches have gained prominence as a framework for understanding comorbidity across mental disorders by identifying shared psychological processes that cut across traditional diagnostic boundaries [20, 45]. Within this perspective, ED and social anxiety symptoms are increasingly conceptualized as partially overlapping expressions of common vulnerabilities rather than as independent syndromes that merely co-occur. Several shared processes have been proposed to account for their co-occurrence, including fear of negative evaluation [17], social appearance anxiety [38, 48], shame [56], and maladaptive forms of perfectionism [55, 74]. However, empirical models integrating multiple transdiagnostic processes simultaneously remain scarce.

Maladaptive perfectionism is typically defined as the tendency to set excessively high personal standards and to be overly concerned about making mistakes or being negatively evaluated by others. It involves harsh self-criticism, fear of failure, and persistent dissatisfaction with one’s performance, even when objectively successful. This construct was first formalized by Frost and colleagues, who distinguished it from adaptive aspects of perfectionism, emphasizing the pathological concern over errors. Recent studies highlight its transdiagnostic relevance, linking maladaptive perfectionism to increased risk and severity of eating disorders and social anxiety [46, 52]. Conceptualized primarily through excessive concern over mistakes and rigid personal standards, maladaptive perfectionism has been consistently associated with greater eating pathology ([6, 12], heightened social anxiety ([23]), and the co-occurrence of both symptom domains [46, 48]. In particular, the concern over mistakes dimension has been directly linked to both anorexia nervosa and bulimia nervosa, highlighting the role of self-critical evaluative processes in eating disorder psychopathology [19, 74], and has been shown to operate as a transdiagnostic feature across restrictive and binge–purge presentations [3]. Individuals characterized by high concern over mistakes tend to overestimate the costs of imperfection and negative evaluation, fostering avoidance, self-criticism, and maladaptive coping strategies that are central to both disorders. Importantly, interventions targeting perfectionistic cognitions—such as exposure to mistakes and modification of rigid standards—have demonstrated reductions in both eating disorder and social anxiety symptoms [35, 62], supporting the transdiagnostic relevance of maladaptive perfectionism as a clinically meaningful intervention target across both eating disorder and social anxiety presentations. Importantly, these associations have also been observed in non-clinical and community samples, where maladaptive perfectionism has been linked to elevated eating disorder symptoms and social anxiety, supporting its relevance across subclinical populations [20, 48].

Another process of increasing interest in transdiagnostic models is psychological inflexibility, particularly experiential avoidance, which has been conceptualized as a short-term emotion regulation strategy that may paradoxically contribute to long-term symptom maintenance. Experiential avoidance refers to attempts to evade, suppress, or control unwanted internal experiences (e.g., thoughts, emotions, bodily sensations), even when such strategies lead to long-term costs [40]. High levels of experiential avoidance have been documented across a wide range of psychopathological conditions [31] and are especially prominent in anxiety disorders [60]. In the context of eating disorders, experiential avoidance has been linked to greater symptom severity and maintenance of disordered eating Behaviours [32, 56], and has been shown to mediate the association between anxiety-related vulnerabilities and eating pathology [22]. Emerging evidence further indicates that experiential avoidance is associated with both restrictive and binge–purge eating behaviours, suggesting a transdiagnostic role in the maintenance of eating disorder symptomatology [32]. In the context of social anxiety, previous research has consistently linked experiential avoidance to greater severity of anxiety symptoms, including social anxiety, where it has been identified as a transdiagnostic vulnerability and maintenance process associated with increased psychopathology and poorer functioning [7, 40, 60, 66]. Similarly as maladaptive perfectionism, experiential avoidance has been associated with both eating disorder and anxiety symptoms in non-clinical samples, further supporting its role as a transdiagnostic process across the continuum of symptom severity [45, 70].

Despite growing recognition of both maladaptive perfectionism and experiential avoidance as transdiagnostic processes, several gaps remain in the literature. First, few studies have examined these processes simultaneously within a single integrative model linking ED and social anxiety symptoms. Second, research has often treated psychological flexibility or inflexibility as a global construct, despite evidence that its subcomponents may relate differentially to specific forms of psychopathology [16, 40]. Measures such as the Personalized Psychological Flexibility Index allow for the examination of experiential avoidance as a distinct dimension with strong psychometric support [1]. Third, most existing studies have focused on unidirectional pathways, leaving the bidirectional nature of ED–social anxiety associations underexplored within process-based models [2, 46].

Given prior evidence suggesting potential sex differences in both eating disorder and social anxiety symptom expression, exploratory analyses were conducted to examine whether the proposed associations were consistent across male and female participants.

Addressing these limitations, the present study adopts a transdiagnostic and cognitive-contextual perspective to test a bidirectional structural model linking eating disorder and social anxiety symptom risk and severity, while simultaneously examining maladaptive perfectionism and experiential avoidance as transdiagnostic processes involved in these associations. Using structural equation modelling in a large sample of young adults, we evaluate (a) the reciprocal relationships between ED and social anxiety symptoms, (b) the direct and indirect associations involving maladaptive perfectionism and experiential avoidance on each symptom domain, and (c) the incremental contribution of these processes relative to models without mediators or with a single transdiagnostic factor. By integrating these processes within a unified structural framework, the present study aims to advance understanding of the shared mechanisms underlying ED–social anxiety comorbidity and to inform the development of more effective, process-focused interventions for young people.

## Method

### Participants

The present study employed a cross-sectional, correlational design conducted in Seville, Spain. Participants were recruited from three universities and four secondary and vocational education centres using non-probabilistic convenience sampling. The target population comprised young adults aged 18–25 years, a developmental period characterized by increased vulnerability to both eating disorder and social anxiety symptomatology.

A total of 890 students initially participated. After data screening and removal of cases with missing or inconsistent responses, the final analytic sample consisted of 705 participants (Mage = 20.02, SD = 1.94). Participants reported their biological sex (male/female). Of these, 338 were women (38%; Mage = 20.21, SD = 1.89), 546 were men (61.3%; Mage = 19.91, SD = 1.97), and a small proportion did not report their sex. An a priori power analysis was not conducted prior to recruitment, as participants were recruited from available educational centres during the data collection period. However, the final sample size exceeded commonly recommended thresholds for structural equation modelling with models of moderate complexity [33].

In the sample, 18.4% of participants scored above the established cut-off for eating disorder risk on the EAT-26 (≥ 20), whereas 58.9% exceeded the threshold for moderate to high social anxiety on the LSAS (≥ 60). These cut-offs were used for descriptive purposes only and were not applied in the structural models, where ED and SA symptom risk were analysed as continuous variables.

Focusing on late adolescence and emerging adulthood is theoretically and clinically justified, as personality traits tend to stabilize during this period [8, 64], which coincides with peak risk for the onset of eating disorder and social anxiety symptoms [61, 69].

## Measures

### Eating disorder symptom risk

Eating disorder symptom risk was assessed using the Eating Attitudes Test–26 (EAT-26) [28], a widely used screening instrument designed to identify disordered eating attitudes and Behaviours. The EAT-26 uses a cutoff score of 20 to indicate elevated risk for eating disorder symptomatology. The instrument includes 6 Likert response options. The total score is obtained by summing the ratings of the 26 items that make up the test: Items 1 and 25 (never = 3 points, almost never = 2 points, sometimes = 1 point, other = 0 points). Remaining items (always = 3 points, almost always = 2 points, often = 1 point, other = 0 points). In the present study, the scale demonstrated excellent internal consistency (Cronbach’s α = 0.90) (test-retest reliability was *r* = .93). The EAT-26 has also been validated in Spanish non-clinical and clinical samples [59, 63], showing adequate reliability and construct validity.

### Maladaptive perfectionism

Maladaptive perfectionism was measured using selected subscales of the Frost Multidimensional Perfectionism Scale (FMPS) [26]. Consistent with prior research, the subscales Concern over Mistakes (assessing excessive fear of errors and sensitivity to negative evaluation) and Personal Standards (assessing rigid, excessively high self-imposed standards) were retained as indicators of maladaptive perfectionism [46, 53, 54, 75]. The FMPS has shown strong psychometric properties in previous studies, and internal consistency in the present sample ranged from α = 0.87 to 0.93 across subscales. Evidence also supports the construct validity and use of the FMPS in both clinical and non-clinical populations, including Spanish-speaking samples [15, 29, 46].

### Experiential avoidance

Experiential avoidance was assessed using the Experiential Avoidance subscale of the Personalized Psychological Flexibility Index (PPFI; [41]), a self-report measure of psychological flexibility and inflexibility. This subscale was used to capture tendencies to avoid unwanted internal experiences. Items are rated on a 7-point Likert-type scale ranging from 1 (strongly disagree) to 7 (strongly agree), with higher scores indicating greater experiential avoidance. The PPFI has demonstrated good psychometric properties in prior research, and recent adaptations support its use in young adult populations [1, 37]. In the present study, internal consistency for the experiential avoidance subscale was acceptable (α = 0.84). Focusing on experiential avoidance as a distinct component of psychological inflexibility is consistent with contemporary conceptualizations emphasizing the multidimensional nature of flexibility-related processes [16].

### Social anxiety symptom risk

Social anxiety symptom risk was measured with the Liebowitz Social Anxiety Scale (LSAS) [50], a 24-item instrument assessing fear and avoidance across social and performance situations. A cut-off score of 60 is commonly used to indicate moderate social anxiety symptom severity. The Spanish version of the LSAS has demonstrated excellent internal consistency, with Cronbach’s α values of 0.95 for the total score, 0.93 for the fear subscale, and 0.91 for the avoidance subscale (Olivares et al., 2005). This validation supports its reliability and construct validity in non-clinical [14] and clinical samples [9].

A detailed description of item content and response formats is provided in Table 1 (see end of manuscript).

**Table 1:** Measures, sample items, and response formats

Instrument	Item no.	Sample item	Response format
EAT-26 [28]	1	I am terrified about being overweight.	7-point Likert scale
	2	I avoid eating even when I am hungry.	7-point Likert scale
	5	I particularly avoid foods high in carbohydrates (e.g., bread, rice, potatoes).	7-point Likert scale
	6	I feel extremely guilty after eating.	7-point Likert scale
	7	I am preoccupied with a desire to be thinner.	7-point Likert scale
	8	I am concerned about having body fat.	7-point Likert scale
	11	I feel that food controls my life.	7-point Likert scale
FMPS (Frost et al. 1990)	4	If I do not set very high standards for myself, I am likely to end up a second-rate person.	7-point Likert scale
	6	It is important to me to be absolutely competent in everything I do.	7-point Likert scale
	11	I set higher goals for myself than most people.	7-point Likert scale
	12	If someone does something better than I do, I feel like a complete failure.	7-point Likert scale
	13	If I fail partly, it is as bad as being a complete failure.	7-point Likert scale
	14	I hate not being the best at everything I do.	7-point Likert scale
	15	I set excessively high goals for myself.	7-point Likert scale
LSAS (Liebowitz 1987)	4	Drinking with others in public places.	0–3 fear intensity
	9	Writing while being observed.	0–3 fear intensity
	10	Meeting new people.	0–3 fear intensity
	11	Urinating in public restrooms.	0–3 fear intensity
	4	Drinking with others in public places.	0–3 avoidance
	8	Working while being observed.	0–3 avoidance
	9	Writing while being observed.	0–3 avoidance
PPFI [41]	1	When I feel stressed while pursuing a goal, I give up.	7-point Likert scale
	2	I am so caught up in my thoughts and feelings that I am unable to pursue my goal.	7-point Likert scale

Items are illustrative examples of those retained for analysis and do not represent the full content of each scale. Response formats correspond to the original instruments; for analytic purposes, all items were recoded to a 7-point Likert scale to ensure comparability across measures. For the LSAS, both fear intensity and avoidance were assessed for the same social situations

EAT-26: Eating Attitudes Test; FMPS: Frost Multidimensional Perfectionism Scale

### Procedure

Inclusion criteria were: (a) age between 18 and 25 years, (b) current enrollment in an educational institution, and (c) completion of the full assessment protocol.

Data collection was conducted in group sessions within classroom settings and lasted approximately 40 min. Prior to participation, the study aims were explained, anonymity and confidentiality were guaranteed, and written informed consent was obtained from all participants. Participants completed the questionnaires in paper-and-pencil format.

Missing data were examined prior to analyses. Cases were excluded when questionnaires showed substantial missing data that prevented reliable scoring of the study measures or when inconsistent response patterns were identified. Formal statistical tests of the missing data mechanism (e.g., MCAR or MAR) were not conducted. Given the relatively large initial sample and the retention of a substantial final sample (*N* = 705), listwise deletion was applied, retaining only complete cases for analysis. This approach is commonly used in structural equation modelling and is considered appropriate when sample size is sufficient and the proportion of missing data is limited, as it is unlikely to substantially bias parameter estimates under these conditions [21, 33].

The study protocol was approved by the Ethics Committee of the University of Seville, and all procedures complied with the ethical standards of the Declaration of Helsinki.

### Data analysis

Statistical analyses were conducted using IBM SPSS Statistics 29.0 and IBM SPSS AMOS 29.0. Preliminary analyses in SPSS included detection of outliers, computation of descriptive statistics, assessment of normality, and exploratory factor analyses (EFA) to refine item sets and examine internal structure. Given the study design and the use of structural equation modelling, the final sample size was considered adequate for stable parameter estimation.

EFA was performed using principal axis factoring with Promax rotation, assuming correlated factors and Kaiser normalization. Items with cross-loadings or factor loadings below 0.60 were removed, following established recommendations for robust factorial solutions [33]. Sampling adequacy was assessed using the Kaiser–Meyer–Olkin (KMO) index and Bartlett’s test of sphericity. Internal consistency was evaluated using Cronbach’s alpha coefficients.

Subsequently, confirmatory factor analysis (CFA) and structural equation modelling (SEM) were conducted in AMOS. Model fit was evaluated using multiple indices, including χ²/df, SRMR, GFI, AGFI, IFI, TLI, CFI, and RMSEA. Composite reliability (CR) and average variance extracted (AVE) were computed to assess construct reliability and convergent validity.

Although univariate normality was observed, multivariate normality was not met (kurtosis = 25.48; [13]). Therefore, bootstrapping procedures with 5,000 resamples and 95% bias-corrected confidence intervals were employed. Overall model fit was additionally evaluated using the Bollen–Stine bootstrap test [10].

### Measurement invariance and additional analyses

Measurement invariance across sex was tested using multigroup SEM, evaluating configural, metric, and scalar invariance for all latent constructs (eating disorder symptom risk, social anxiety symptom risk, maladaptive perfectionism, and experiential avoidance). Invariance was assessed using χ² difference tests and changes in CFI and RMSEA.

Discriminant validity was examined using the Fornell–Larcker criterion [25] and the heterotrait–monotrait ratio (HTMT). Common method bias was assessed using Harman’s single-factor test. Multicollinearity between maladaptive perfectionism and experiential avoidance was evaluated using bivariate correlations and collinearity diagnostics (variance inflation factor, tolerance, and condition index).

Following validation of the measurement model, alternative structural models were estimated to test direct, indirect, and total effects. In theoretically justified cases, correlated error terms between items of the same construct were permitted based on modification indices and conceptual rationale [33, 72]. Indirect effects were evaluated using bootstrap confidence intervals.

## Results

### Preliminary analyses and exploratory factor structure

Prior to testing the structural models, data were screened for missing values, outliers, and distributional assumptions. After data cleaning, the final analytic sample comprised 705 participants. Exploratory factor analyses were conducted to refine item sets and examine the internal structure of the measures included in the model. In the present analyses, ED and SA symptom risk were operationalised as continuous latent variables derived from self-report measure (EAT-26 and LSAS, respectively), with higher scores indicating greater symptom severity. No clinical cut-off scores were applied, as the study aimed to examine dimensional associations across the full range of symptomatology.

Sampling adequacy was satisfactory (KMO = 0.868), and Bartlett’s test of sphericity was significant, χ²(210) = 6713.73, *p* < .001, supporting the suitability of the data for factor analysis. The resulting solution yielded four factors corresponding to eating disorder symptom risk, social anxiety symptom risk, maladaptive perfectionism, and experiential avoidance, which together explained 61.64% of the total variance.

Items retained for eating disorder symptom risk loaded coherently on a single factor (α = 0.89). For maladaptive perfectionism, items reflecting concern over mistakes and personal standards showed adequate factor loadings and internal consistency, whereas items reflecting doubts about actions failed to meet loading criteria and were excluded. The experiential avoidance factor, assessed using items from the PPFI, showed acceptable internal consistency (α = 0.73). Social anxiety symptom risk items loaded clearly on their respective factor (α = 0.85). Detailed factor loadings and reliability indices are presented in Table 2.

**Table 2:** Factor loadings, reliability, and convergent validity of the measurement model

Construct	Item	EFA loading (component)	CFA loading	Cronbach’s α	CR	AVE
Eating disorder symptom risk	Item 1	0.856 (C1)	0.651	0.888	0.886	0.528
	Item 2	0.793 (C1)	0.747			
	Item 3	0.837 (C5)	0.787			
	Item 4	0.787 (C2)	0.822			
	Item 5	0.857 (C2)	0.620			
	Item 6	0.842 (C2)	0.764			
	Item 7	0.749 (C2)	0.669			
Social anxiety symptom risk	Item 1	0.874 (C4)	0.907	0.847	0.845	0.528
	Item 2	0.829 (C3)	0.768			
	Item 3	0.904 (C4)	0.574			
	Item 4	0.825 (C3)	0.601			
	Item 5	0.881 (C3)	0.734			
Experiential avoidance	Item 1	0.856 (C1)	0.629	0.727	0.752	0.611
	Item 2	0.793 (C1)	0.909			
Personal standards	Item 1	0.809 (C3)	0.809	0.796	0.798	0.502
	Item 2	0.774 (C3)	0.774			
	Item 3	0.576 (C4)	0.576			
	Item 4	0.650 (C4)	0.650			
Concern over mistakes	Item 1	0.792 (C1)	0.692	0.802	0.811	0.589
	Item 2	0.762 (C1)	0.767			
	Item 3	0.799 (C5)	0.837			

Component numbers from the EFA are shown in parentheses. All retained indicators met recommended thresholds (factor loadings ≥ 0.50; Cronbach’s α and CR ≥ 0.70; AVE ≥ 0.50)

EFA: exploratory factor analysis (principal axis factoring, Promax rotation). CFA: confirmatory factor analysis. Cronbach’s α: internal consistency reliability; CR: composite reliability; AVE: average variance extracted

### Measurement model

The measurement model demonstrated adequate fit to the data, with all fit indices meeting recommended criteria (Table 3). All factor loadings were statistically significant and exceeded accepted thresholds, and internal consistency and composite reliability indices were satisfactory across constructs (Table 2). Convergent validity was supported by average variance extracted values above recommended cut-offs, and discriminant validity was confirmed using the Fornell–Larcker criterion [25] and HTMT ratios (Table 4).

**Table 3:** Fit indices for the measurement model

Fit index	Value
χ² (CMIN)	476.83
df	175
χ²/df	2.73
CFI	0.954
TLI	0.945
IFI	0.954
NFI	0.930
RMSEA	0.049
90% CI RMSEA	[0.044, 0.055]
SRMR	0.050
PCLOSE	0.553

χ²: chi-square; df: degrees of freedom; CFI: Comparative Fit Index; TLI: Tucker–Lewis Index; IFI: Incremental Fit Index; NFI: Normed Fit Index; RMSEA: Root Mean Square Error of Approximation; SRMR: Standardised Root Mean Square Residual; CI: confidence interval; PCLOSE: probability of close fit (RMSEA ≤ 0.05)

**Table 4:** Discriminant Validity Matrix (Fornell–Larcker Criterion and HTMT Ratios)

Construct	ED risk	SA risk	Experiential avoidance	Personal standards	Concern over mistakes
ED risk	**0.727**				
SA risk	0.134 (0.268)	**0.727**			
Experiential avoidance	−0.183 (0.335)	−0.167 (0.339)	**0.782**		
Personal standards	0.086 (0.170)	−0.015 (0.099)	−0.062 (0.150)	**0.709**	
Concern over mistakes	0.186 (0.342)	0.132 (0.258)	−0.231 (0.389)	0.364 (0.703)	**0.767**

Values on the diagonal (in bold) represent the square roots of the average variance extracted (AVE) for each construct. Values below the diagonal represent inter-construct correlations. Values in parentheses correspond to heterotrait–monotrait (HTMT) ratios. All HTMT values were below the recommended threshold of 0.85, indicating adequate discriminant validity among constructs

ED risk: eating disorder symptom risk; SA risk: social anxiety symptom risk

**Table 5:** Standardised direct and indirect associations for hypothesised paths

Hypothesis	Path	Effect type	β	95% BCa CI	*p*
H1a	ED risk → SA risk	Direct	0.167	[0.068, 0.264]	0.003
H1b	SA risk → ED risk	Direct	0.146	[0.068, 0.236]	0.001
H2	Perfectionism → ED risk	Direct	0.188	[0.071, 0.293]	0.004
H2i	Perfectionism → ED risk	Indirect	0.013	[0.003, 0.033]	0.012
H3	Perfectionism → SA risk	Direct	0.107	[0.022, 0.167]	0.024
H3i	Perfectionism → SA risk	Indirect	0.030	[0.011, 0.068]	0.008
H4	Experiential avoidance → ED risk	Direct	−0.228	[− 0.352, − 0.115]	< 0.001
H4i	Experiential avoidance → ED risk	Indirect	0.058	[0.028, 0.110]	< 0.001
H5	Experiential avoidance → SA risk	Direct	−0.196	[− 0.309, − 0.092]	< 0.001
H5i	Experiential avoidance → SA risk	Indirect	0.078	[0.035, 0.142]	< 0.001

Note. Standardised estimates (β) are reported. Bias-corrected and accelerated (BCa) 95% confidence intervals were estimated using bootstrapping with 5,000 resamples

ED risk: eating disorder symptom risk; SA risk: social anxiety symptom risk

### Measurement invariance across sex

Measurement invariance across sex was examined using multigroup CFA. The configural model showed acceptable fit, indicating a comparable factor structure across women and men. Imposing equality constraints on factor loadings (metric invariance) resulted in a statistically significant χ² difference; however, changes in incremental fit indices (ΔCFI and ΔRMSEA) were below recommended cut-offs, supporting metric invariance.

Scalar invariance was not fully supported, as constraining item intercepts led to a significant deterioration in model fit. Given that the primary aim was to examine structural associations rather than latent mean differences, metric invariance was considered sufficient to allow meaningful comparison of relationships among constructs across sex.

### Structural models and hypothesis testing

Two alternative structural models were estimated to examine the bidirectional associations between eating disorder (ED) and social anxiety (SA) symptom risk and the indirect associative roles of maladaptive perfectionism and experiential avoidance (see Table 5 for model fit indices).

**Fig. 1:**
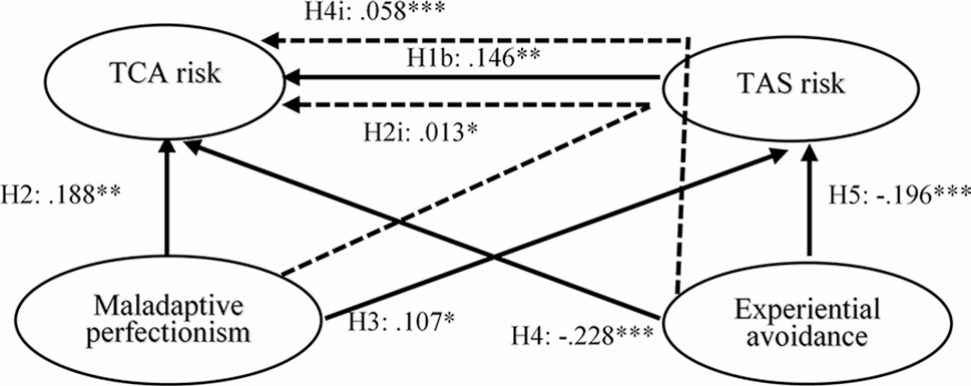
Structural model A: direct and indirect effects of maladaptive perfectionism and experiential avoidance on eating disorder and social anxiety symptom risk. ED: eating disorder symptom risk; SA: social anxiety symptom risk. Standardised path coefficients (β) are shown. Solid lines indicate statistically significant paths (*p* < .05; *p* < .01; *p* < .001)

**Model A**, in which ED symptom risk predicted SA symptom risk, demonstrated adequate fit to the data. As illustrated in Fig. 1, ED symptom risk was positively associated with SA symptom risk, supporting Hypothesis 1a. Maladaptive perfectionism and experiential avoidance showed significant direct and indirect associations with both ED and SA symptom risk, with experiential avoidance displaying opposing direct and indirect effects. All hypothesised associations were statistically significant and in the expected directions (Table 6).

**Table 6:** Fit indices for alternative structural models

Model	χ²/df	CFI	TLI	NFI	RMSEA (90% CI)	SRMR
Model A (ED → SA)	2.90	0.949	0.939	0.925	0.052 [0.047, 0.057]	0.078
Model B (SA → ED)	2.70	0.955	0.946	0.930	0.049 [0.044, 0.054]	0.057

χ²/df: chi-square to degrees of freedom ratio; CFI: Comparative Fit Index; TLI: Tucker–Lewis Index; NFI: Normed Fit Index; RMSEA: Root Mean Square Error of Approximation; CI: confidence interval; SRMR: Standardised Root Mean Square Residual

Model A specifies eating disorder symptom risk predicting social anxiety symptom risk

Model B specifies social anxiety symptom risk predicting eating disorder symptom risk

**Fig. 2:**
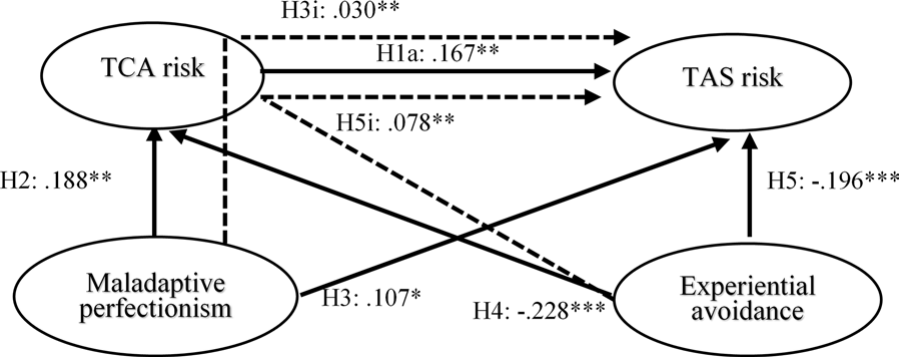
Structural model B: bidirectional associations between social anxiety and eating disorder symptom risk involving transdiagnostic processes. ED: eating disorder symptom risk; SA: social anxiety symptom risk. Standardised path coefficients (β) are shown. Solid lines indicate statistically significant paths (*p* < .05; *p* < .01; *p* < .001)

**Model B**, specifying SA symptom risk as a predictor of ED symptom risk, also demonstrated good fit and a pattern of associations comparable to Model A (Fig. 2). SA symptom risk significantly predicted ED symptom risk, supporting Hypothesis 1b. Fit indices indicated that Model B showed slightly superior fit across incremental and absolute indices; however, the pattern of direct and indirect associations was highly consistent across models. Standardised effects are reported in Table 6, and comparisons among alternative structural models are presented in Table 7.

**Table 7:** Comparison of alternative structural models

Model	χ² (df)	χ²/df	CFI	TLI	RMSEA (90% CI)	AIC	BIC
Baseline model	589.34 (178)	3.31	0.937	0.926	0.057 [0.052, 0.062]	695.34	936.92
Perfectionism-only model	564.09 (176)	3.21	0.941	0.930	0.056 [0.051, 0.061]	674.09	924.79
Experiential avoidance–only model	507.57 (176)	2.88	0.950	0.940	0.052 [0.047, 0.057]	617.57	868.27
Full model (perfectionism + experiential avoidance)	491.48 (174)	2.83	0.952	0.942	0.051 [0.046, 0.056]	605.48	865.30

Lower AIC and BIC values indicate better model fit while accounting for model complexity

χ²/df: chi-square to degrees of freedom ratio; CFI: Comparative Fit Index; TLI: Tucker–Lewis Index; RMSEA: Root Mean Square Error of Approximation; CI: confidence interval; AIC: Akaike Information Criterion; BIC: Bayesian Information Criterion

### Comparison of alternative structural models

Comparisons among alternative structural models indicated that the full model including both maladaptive perfectionism and experiential avoidance provided the best balance between fit and parsimony (Table 7).

## Discussion

The present study examined the bidirectional association between eating disorder (ED) and social anxiety (SA) symptom risk and severity from a transdiagnostic perspective, focusing on maladaptive perfectionism and experiential avoidance as indirect associative processes. Findings indicate reciprocal associations between ED and SA symptoms and highlight maladaptive perfectionism and experiential avoidance as relevant processes linking these symptom domains, consistent with prior transdiagnostic and comorbidity research [20, 46, 73]. These results extend previous work by integrating cognitive and contextual processes within a unified structural framework.

### Bidirectional association between eating disorder and social anxiety symptoms

Consistent with prior longitudinal, prospective, and network-based research, ED and SA symptoms were reciprocally associated: eating disorder symptom risk predicted social anxiety risk, and social anxiety risk predicted eating disorder symptoms [42, 46, 47, 52]. Network analyses suggest these symptoms operate within overlapping vulnerability systems rather than as isolated clinical entities [38]. This reciprocal association highlights the potential for social anxiety to exacerbate eating pathology and vice versa, reinforcing the clinical relevance of comorbid ED–SA presentations [11].

From a clinical perspective, such bidirectionality helps explain the greater severity, chronicity, and poorer outcomes observed in comorbid ED–SA cases [5, 71]. High rates of anxiety comorbidity in EDs have important implications for functional impairment and treatment response, emphasizing the need for integrated assessment and intervention targeting shared psychological processes such as evaluative concerns, avoidance, and maladaptive coping [57].

### Maladaptive perfectionism as a transdiagnostic risk process

Maladaptive perfectionism emerged as a consistent transdiagnostic risk factor for both ED and SA symptoms. Higher levels of concern over mistakes and rigid personal standards were associated with increased symptom risk in both domains, with significant direct and indirect effects across models. These findings are in line with a substantial body of literature identifying perfectionism as a central vulnerability for eating disorders [4, 12] and social anxiety [23], as well as for their co-occurrence [46].

The presence of both direct and indirect effects suggests that maladaptive perfectionism may be involved through multiple pathways, rather than functioning solely as a distal background factor. Excessive concern over mistakes and rigid standards may heighten fear of negative evaluation, promote self-criticism, and foster avoidance-based coping strategies that are central to both ED and SA symptomatology [18, 55]. Interventions targeting perfectionism—such as exposure to mistakes and modification of rigid standards—have been associated with reductions in both social anxiety and eating disorder symptoms, supporting the clinical relevance of maladaptive perfectionism as a process-focused intervention target [35, 62]. Addressing perfectionism may improve outcomes across comorbid and subthreshold ED–SA presentations, highlighting its value in clinical practice [48].

### Experiential avoidance and inconsistent indirect pathways

Experiential avoidance showed a more complex pattern of associations with ED and SA symptom risk. This finding is theoretically consistent with contextual and acceptance-based models, which conceptualize experiential avoidance as a short-term emotion regulation strategy that provides immediate relief but contributes to longer-term symptom maintenance [40, 45]. These indirect associations should be interpreted as explanatory pathways within a cross-sectional structural model, rather than as evidence of temporal or causal mediation.

Previous research has consistently linked experiential avoidance to greater severity of anxiety symptoms [60] and eating disorder psychopathology [22, 32]. The present findings help reconcile mixed results in the literature by suggesting that experiential avoidance may exert opposing effects depending on the pathway considered. Avoidance of distressing internal experiences may temporarily reduce anxiety or discomfort related to eating or social situations, yet indirectly increase vulnerability by reinforcing rigid coping strategies, limiting corrective experiences, and maintaining fear-based responding [31, 56].

Importantly, targeting experiential avoidance through process-focused, acceptance-based interventions, such as Acceptance and Commitment Therapy (ACT), has shown efficacy in reducing both eating disorder and anxiety symptoms, highlighting its clinical relevance [24, 34, 39, 43]. Addressing experiential avoidance alongside maladaptive perfectionism may improve outcomes in comorbid or subthreshold ED–SA presentations, supporting integrative, transdiagnostic interventions [73].

### Incremental contribution of transdiagnostic processes

Model comparisons indicated that incorporating transdiagnostic processes substantially improved explanatory power relative to a baseline model including only the direct association between ED and SA symptom risk. Experiential avoidance accounted for a larger proportion of incremental model improvement than maladaptive perfectionism when considered independently, and the combined model including both processes provided the best balance between fit and parsimony. These findings align with process-based models of psychopathology emphasizing the convergence of multiple shared mechanisms rather than reliance on a single underlying vulnerability [20, 65].

This highlights the potential utility of integrative, process-focused interventions that simultaneously target evaluative concerns and maladaptive emotion regulation strategies, offering a more comprehensive approach to reducing symptoms across both domains [73].

### Clinical implications

The findings have important clinical implications. First, they underscore the need for integrated assessment of eating disorder (ED) and social anxiety (SA) symptoms, particularly in young adults, given their bidirectional associations and shared psychological processes [61]. Second, the results highlight the relevance of transdiagnostic, process-focused approaches that address both maladaptive perfectionism and experiential avoidance. Cognitive–behavioural strategies aimed at reducing rigid evaluative standards and excessive concern over mistakes may be meaningfully integrated with contextual interventions, such as exposure, acceptance, and values-based action, to reduce avoidance and promote psychological flexibility [62, 73]. Importantly, interventions specifically targeting perfectionism, such as cognitive–behavioural perfectionism-focused therapy, and acceptance-based interventions addressing experiential avoidance, including Acceptance and Commitment Therapy (ACT), have shown efficacy in reducing eating disorder symptoms and associated psychopathology in both clinical and subthreshold populations [39, 44, 68].

From a clinical standpoint, these findings suggest that early assessment of maladaptive perfectionism and experiential avoidance may help identify individuals at increased risk of comorbid eating disorder and social anxiety symptom presentations. In practical terms, incorporating brief screening of evaluative concerns and avoidance patterns into routine assessment may improve case formulation and support more targeted intervention planning. Additionally, integrating strategies that simultaneously address rigid evaluative standards and avoidance-based coping may enhance treatment responsiveness, particularly in young adults presenting with overlapping symptom profiles.

Such integrative approaches may complement existing treatments and inform assessment and formulation for individuals presenting with comorbid or subthreshold ED and SA symptoms, for whom disorder-specific interventions may be insufficiently comprehensive. This perspective is consistent with growing evidence emphasising the importance of targeting shared psychological mechanisms in eating disorders, particularly in complex and comorbid presentations [51].

### Limitations and future directions

Several limitations of the present study should be acknowledged. First, the cross-sectional design precludes causal inferences regarding the temporal ordering of eating disorder (ED) and social anxiety (SA) symptom risk and the indirect associative roles of maladaptive perfectionism and experiential avoidance. Although the bidirectional structural models were theoretically grounded and statistically supported, longitudinal and prospective designs are required to determine whether these transdiagnostic processes operate as antecedents, consequences, or maintaining factors over time. This limitation is common in transdiagnostic research on ED and anxiety comorbidity and highlights the need for designs capable of capturing developmental and reciprocal dynamics [46, 69].

Second, all constructs were assessed using self-report measure, which may introduce biases related to social desirability, shared method variance, and retrospective reporting. Although several procedural and statistical steps were taken to mitigate common method bias, future research would benefit from multimethod assessment strategies, including structured clinical interviews, Behavioural or experimental tasks, and ecological momentary assessment approaches. Such strategies may be particularly informative for processes such as experiential avoidance and perfectionism, which may operate partly outside of conscious awareness [22]. Anthropometric variables such as body mass index (BMI) and weight suppression were not assessed, which may represent relevant covariates in the study of eating disorder symptomatology. Future research should incorporate these measures to better account for their potential confounding effects. Furthermore, a proportion of cases were excluded due to missing or inconsistent responses, and listwise deletion was applied. Although this approach is commonly used in structural equation modelling with sufficiently large samples, it may have introduced some degree of sample bias by retaining only complete cases. Future studies could consider alternative methods for handling missing data, such as multiple imputation or full information maximum likelihood, to examine the robustness of the findings.

Third, the sample consisted of non-clinical young adults recruited through convenience sampling from educational settings. While this approach is appropriate for examining symptom risk and transdiagnostic processes during a critical developmental period, it limits the generalizability of the findings to clinical populations, treatment-seeking individuals, older age groups, and culturally diverse contexts. Replication in clinical samples and across developmental stages is therefore necessary to establish the broader applicability of the proposed model [42]. Indeed, the predominance of male participants in our sample may limit the generalizability of the findings to female populations, in which most research on eating disorders has been conducted [27]. Future research should aim to replicate these findings in samples with higher proportions of female participants and/or conduct sex-stratified analyses to examine potential differences in transdiagnostic processes across genders. In addition, standard self‑report eating disorders questionnaires were originally developed and validated predominantly in female samples and may not fully capture the male experience of disordered eating, potentially underestimating symptom levels or presenting measurement bias in men [67, 76]. Moreover, gender identity was not assessed, which may be considered in future research.

Fourth, although item reduction and the use of selected subscales enhanced parsimony and model fit, this approach may have constrained the breadth of the constructs assessed. Both maladaptive perfectionism and experiential avoidance are multidimensional processes, and future studies should examine whether additional dimensions—such as doubts about actions, acceptance, or values-based action—exhibit differential associations with ED and SA symptom trajectories.

The present model focused on two theoretically relevant transdiagnostic processes but did not include other mechanisms that have been implicated in ED–SA comorbidity. Processes such as intolerance of uncertainty, shame, social appearance anxiety, and fear of negative or positive evaluation may further elucidate shared pathways between these symptom domains and should be incorporated into future integrative models [36, 38]. Additionally, the present study did not differentiate between specific ED diagnoses (e.g., anorexia nervosa vs. bulimia nervosa), which may be relevant given evidence that contributing and maintaining factors can vary across restrictive and binge/purge presentations [12, 69]. Future research should examine whether the transdiagnostic processes identified here operate similarly across different ED subtypes.

Future studies using longitudinal and experimental designs are needed to clarify temporal ordering and causal pathways, and intervention-based designs to clarify the temporal dynamics among ED symptoms, SA symptoms, maladaptive perfectionism, and experiential avoidance. Such designs would allow for formal tests of indirect pathways over time and the examination of reciprocal feedback loops across developmental stages. In addition, randomized controlled trials and process-focused intervention studies are needed to determine whether targeting maladaptive perfectionism and experiential avoidance leads to concurrent reductions in both ED and SA symptoms.

Finally, future investigations may benefit from complementing structural equation modelling with alternative analytic approaches, such as network analysis or dynamic systems modelling, to better capture the complex and potentially nonlinear interactions among symptoms and transdiagnostic processes. Together, these directions may advance a more precise and clinically actionable understanding of the mechanisms underlying eating disorder and social anxiety comorbidity.

## Conclusions

The present study identifies a robust bidirectional association between eating disorder and social anxiety symptom risk in young adults and highlights maladaptive perfectionism and experiential avoidance as relevant transdiagnostic processes associated with this comorbidity. By integrating cognitive and contextual processes within a unified structural framework, the findings advance current understanding of how shared vulnerabilities may be linked to the co-occurrence of eating disorder and social anxiety symptoms.

Maladaptive perfectionism, characterized by rigid personal standards and heightened concern over mistakes, emerged as a consistent transdiagnostic process associated with both symptom domains, showing direct and indirect associations. Experiential avoidance showed a more complex pattern, suggesting short-term relief alongside longer-term vulnerability through indirect pathways. Together, these results underscore the importance of considering multiple transdiagnostic mechanisms simultaneously rather than in isolation.

From a clinical perspective, the findings support the value of process-focused, transdiagnostic interventions that address both maladaptive perfectionism and experiential avoidance. Targeting these processes may complement existing interventions and inform process-focused treatment approaches for individuals presenting with co-occurring or subthreshold eating disorder and social anxiety symptoms, particularly during emerging adulthood.

Overall, this study contributes to a growing body of research emphasizing shared psychological processes in psychopathology and supports the continued development of integrative, transdiagnostic models to better understand the co-occurrence of eating disorder and social anxiety symptoms.

## Data Availability

The datasets used and/or analysed during the current study are available from the corresponding author on reasonable request.

## Abbreviations

ED: Eating disorder
SA: Social anxiety
SAD: Social anxiety disorder
ED–SA: Eating disorder–social anxiety (comorbidity)
R_TCA: Eating disorder symptom risk
R_TAS: Social anxiety symptom risk
EVI: Experiential avoidance
EST_P: Personal standards (perfectionism)
P_E: Concern over mistakes (perfectionism)
SEM: Structural equation modeling
CBT: Cognitive–behavioural therapy
CI: Confidence interval
BCa: Bias-corrected and accelerated (bootstrap confidence intervals)
